# Functional analysis of tomato CHIP ubiquitin E3 ligase in heat tolerance

**DOI:** 10.1038/s41598-021-81372-8

**Published:** 2021-01-18

**Authors:** Yan Zhang, Xiaodong Lai, Siqing Yang, Huan Ren, Jingya Yuan, Huanchun Jin, Chengchen Shi, Zhibing Lai, Gengshou Xia

**Affiliations:** 1 Department of Landscape and Horticulture, Ecology College, Lishui University, Lishui, 323000 Zhejiang China; 2 National Key Laboratory of Crop Genetic Improvement, Huazhong Agricultural University, Wuhan, 430070 China

**Keywords:** Genetics, Plant sciences

## Abstract

Plants have evolved genetic and physiological mechanisms to mitigate the adverse effects of high temperature. CARBOXYL TERMINUS OF THE HSC70-INTERACTING PROTEINS (CHIP) is a conserved chaperone-dependent ubiquitin E3 ligase that targets misfolded proteins. Here, we report functional analysis of the *SlCHIP* gene from tomato (*Solanum lycopersicum*) in heat tolerance. *SlCHIP* encodes a CHIP protein with three tandem tetracopeptide repeat (TPR) motifs and a C-terminal U box domain. Phylogenetic analysis of CHIP homologs from animals, spore-bearing and seed plants revealed a tree topology similar to the evolutionary tree of the organisms. Expression of *SlCHIP* was induced under high temperature and was also responsive to plant stress hormones. Silencing of *SlCHIP* in tomato reduced heat tolerance based on increased heat stress symptoms, reduced photosynthetic activity, elevated electrolyte leakage and accumulation of insoluble protein aggregates. The accumulated protein aggregates in *SlCHIP*-silenced plants were still highly ubiquitinated, suggesting involvement of other E3 ligases in ubiquitination. *SlCHIP* restored the heat tolerance of Arabidopsis *chip* mutant to the wild type levels. These results indicate that tomato SlCHIP plays a critical role in heat stress responses most likely by targeting degradation of misfolded proteins that are generated during heat stress.

## Introduction

Global warming severely threatens crop productivity worldwide. High temperature (heat stress) affects plant vegetative and reproductive growth, and results in decreased productivity and quality of crop products^1,2^. Plants respond to heat stress by deploying a suite of complex events, comprising various signaling pathways, changes in expression of a battery of genes in different regulatory networks, and a cascade of physiological and biochemical processes^3,4^. As the terminal components of high temperature signal transduction, heat shock transcription factors (HSFs) trigger the transcription of heat-responsive genes encoding heat shock proteins (HSPs) and other heat-protective proteins^5^. In plants, both HSF and HSP proteins are encoded by gene families and play critical roles in heat-tolerance by regulating disparate aspects of heat stress responses^6–10^. Genes encoding HSPs and HSFs have been identified and analyzed in many plants including wheat, maize^11–13^, peony, tall fescue, alfalfa, pepper, mung bean and other crops to be vital in plant heat tolerance^14–19^. HSFs, HSPs and other factors associated with plant heat responses modulate cellular and molecular processes that ultimately impact heat tolerance at the whole plant level.

At the cellular level, HSFs and HSPs alleviate the negative effects of heat stress on membrane fluidity, photosynthesis and oxidative homeostasis by boosting chlorophyll content and photosynthetic rate, increasing the activities of antioxidant enzymes, and up or down-regulating specific cellular and biochemical constituents. Antioxidant enzymes including catalases (CAT), peroxidase (POX), ascorbate peroxidases (APX), and superoxide dismutases (SOD) increase in response to heat stress. Similarly heat stress response elevates cellular proline and soluble sugars levels but reduces the levels of malonaldehyde (MDA), reactive oxygen species (ROS) content and relative electrolytic leakage (REL)^12–19^. Apart from HSFs, other types of transcription factors also play important roles in plant heat responses. For example, overexpression of Arabidopsis WRKY30 in transgenic wheat plants elevated contents of chlorophyll, water, prolines, soluble proteins, soluble sugars, and increased antioxidant enzymes activities under heat and drought stress^20^. In rice, a loss-of function mutant for a MYB family transcription factor displayed increased heat tolerance associated with elevated levels of CAT and SOD enzyme activity, total soluble sugar and MDA^21^. Moreover, overexpression of microRNA319d from *Solanum habrochaites* heightened CAT and SOD enzyme activity, chlorophyll content and Fv/Fm values but decreased the levels of REL, MDA and ROS under temperature stress^22^.

At the molecular level, HSPs act as molecular chaperons to control the accumulation of misfolded or damaged proteins generated during heat stress by promoting their folding and refolding or targeting ubiquitination-mediated degradation by autophagy and 26S proteasome system^23–27^. Notably, two proteins from Arabidopsis, AtNBR1 (NEIGHBOR OF BRCA1) and AtCHIP (CARBOXYL TERMINUS OF THE HSC70-INTERACTING PROTEINS), have been shown to play critical roles in targeting degradation of heat-denatured proteins by autophagy and 26S proteasome during heat stress responses. AtNBR1 is an autophagy cargo adaptor that mediates selective autophagy of ubiquitinated damaged protein aggregates caused by heat stress^28^. The AtCHIP E3 ubiquitin ligase acts as a Hsp70 co-chaperone and protects against heat stress-induced proteotoxicity. Genetic analysis indicated that AtCHIP and AtNBR1 function additively in promoting plant heat stress tolerance^29^.

In plants, E3 ubiquitin ligases are divided into four primary categories based on their subunit domain characteristics and mode of action: HECT (Homologous to E6-associated protein Carboxyl Terminus), RING (Really Interesting New Gene), U-box, and CRLs (Cullin-RING ligases)^30,31^. Ubiquitin E3 ligases are involved in regulation of plant growth, development and responses to biotic and abiotic stress^31,32^. Interestingly, there are more than one thousand loci encoding E3 ubiquitin ligases in Arabidopsis^32^. In Arabidopsis, besides the AtCHIP U-box E3 ubiquitin ligase, the E3 ubiquitin ligase MPSR1 also plays a role during the early stage of heat stress response^33^. In rice, a RING type E3 ubiquitin ligase, OsHIRP1, positively regulates plant responses to heat stress^34^.

Despite many years of research, the genetic and molecular basis of plant heat-tolerance mechanisms is still not fully understood, particularly on the extent of conservation and variation of these mechanisms among different plant species^1^. In the present study, we report functional analysis of the homolog of the CHIP E3 ligase from tomato (*Solanum lycopersicum*), SlCHIP, in plant heat stress responses. We analyzed the expression of *SlCHIP* under high temperature and in response to plant stress hormones jasmonic acid (JA), salicylic acid (SA) and abscisic acid (ABA). To address directly the role of SlCHIP in tomato heat tolerance, we down-regulated its expression using virus-induced gene silencing (VIGS) in tomato and comprehensively analyzed the impacts through assessment of heat stress symptoms and other physiological and biochemical parameters including accumulation and ubiquitination of insoluble protein aggregates. The *SlCHIP* gene has also been transformed into Arabidopsis *chip* mutant to determine its ability to restore the heat-tolerant phenotype. The results from the study provide important new insights not only into the role of the conserved chaperone-dependent ubiquitin E3 ligase in plant heat tolerance but also into the possible mechanisms by which the CHIP protein contributes to protein quality control during plant responses to heat stress.

## Results

### Identification and sequence analysis of tomato *SlCHIP*

In order to identify the CHIP homologs in tomato, we conducted BLAST searches against sequenced tomato genome using Arabidopsis CHIP protein sequence as query. To verify a tomato protein as a CHIP homolog, we relied not only on the sequence similarity to Arabidopsis CHIP but also on the presence of both the TPR motifs and U-box domain. Using these criteria, we identified a single tomato gene (Solyc06g083150) encoding a CHIP homolog, which was named *SlCHIP*. Several additional tomato genes encode proteins with high sequence similarity to some parts of Arabidopsis and tomato CHIP proteins but are not considered to be CHIP homologs because they contain only TPR motifs or U box domain. One such gene is Solyc09g082540 that encodes a protein containing three TPR motifs at the N-terminus but lacks the U-box domain. This TPR gene, named *SlTPR28*, was included in the study as a control.

**Figure 1:**
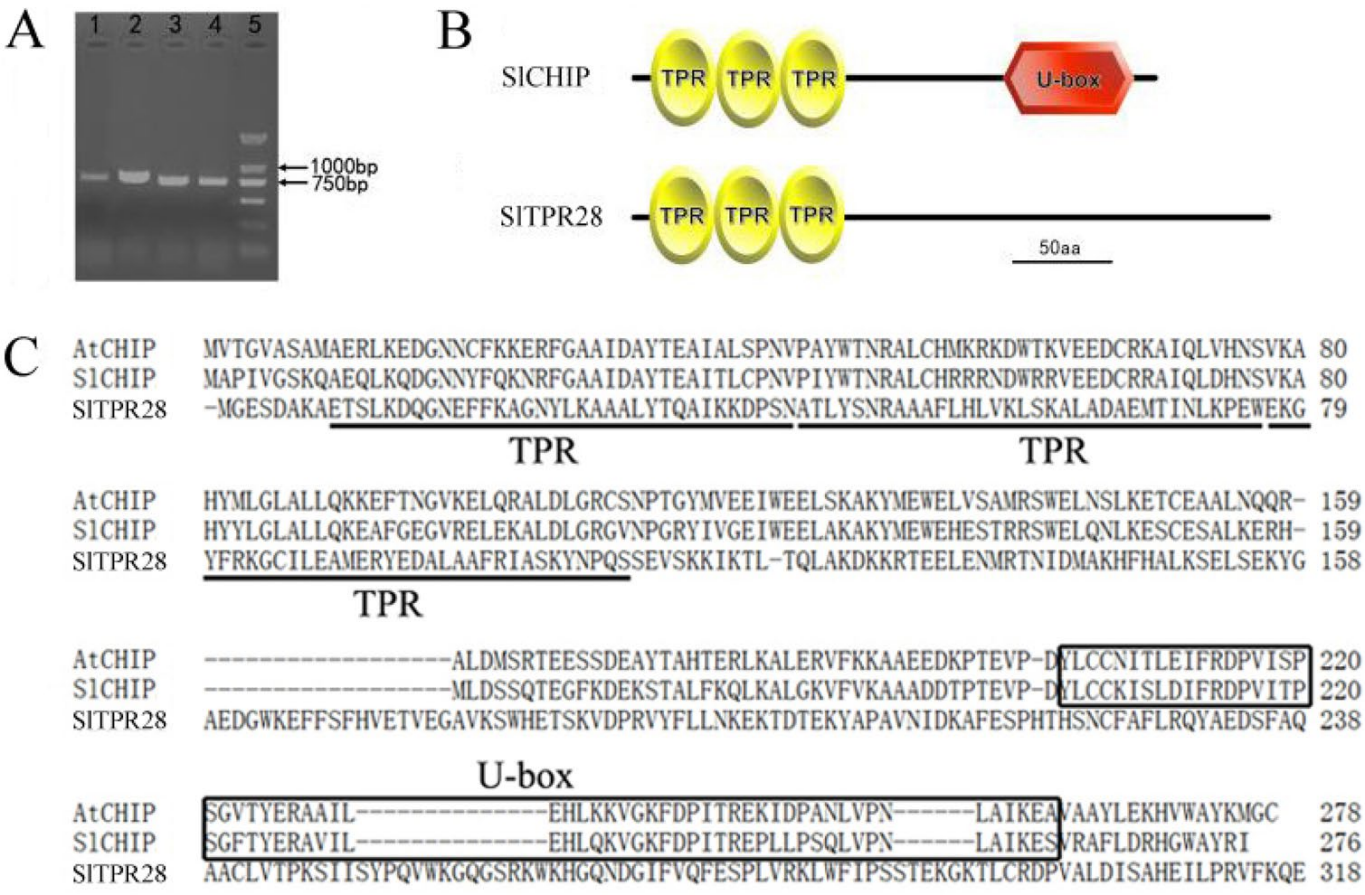
Tomato *SlCHIP* and *SlTPR28* genes and encoded proteins. (**A**) PCR products of *SlTPR28* (lanes 1 and 2) and *SlCHIP* (lanes 3 and 4) coding sequences. (**B**) The TPR motifs and U-box domain in predicted SlCHIP and SlTPR28 proteins. (**C**) Alignment of protein sequences of AtCHIP, SlCHIP and SlTPR28. The three TPR motifs were underlined and the U-box domain was highlighted by a rectangular box.

Using gene-specific primers, we PCR-amplified the coding sequences of both *SlCHIP* and *SlTPR28* using cDNA from tomato cultivar Zheza809 as templates (Fig. 1A). Based on the coding sequence and genome annotation, tomato *SlCHIP* gene contains eight exons and seven introns, which are identical to Arabidopsis *AtCHIP* gene. *SlCHIP* and *SlTPR28* encode two proteins of 276 and 318 amino acids, respectively. The similarity between the deduced SlCHIP and SlTPR28 proteins is 23.9%. SlCHIP shares 68.8% amino acid identity with AtCHIP protein. The deduced SlCHIP protein contains three TPR motifs and one U-box domain based on SMART analysis (http://smart.embl-heidelberg.de/). However, SlTPR28 protein contains only three TPR motifs but no U-box domain (Fig. 1B,C).

### Phylogenetic analysis of SlCHIP homologs from different organisms

CHIP ubiquitin E3 ligases have been identified in all eukaryotic organisms. In contrast to the CHIP proteins from animals, which have been well characterized, there have been only a few studies on plant CHIP proteins, exclusively in Arabidopsis. Therefore, we searched the sequenced genomes of representative plants along the evolutionary tree for genes encoding CHIP homologs, again using the criteria of high sequence similarity and presence of both the TPR motifs and the U-box domain. Using these criteria, we identified 14 CHIP-encoding genes from 10 spore-bearing and seed plants. Like animals, most plants contain a single gene encoding the chaperone-dependent U box E3 ubiquitin ligase. However, some plants including the spreading earthmoss (*Physcomitrella patens*), maize (*Zea mays*), purple false brome (*Brachypodium distachyon*) and soybean (*Glycine max*) contain two genes encoding CHIP proteins. Maize, soybean and purple false brome are known to be polyploid plants that have gone genome duplications during their evolutionary history, which could account for the presence of more than one *CHIP* genes in their genomes.

**Figure 2:**
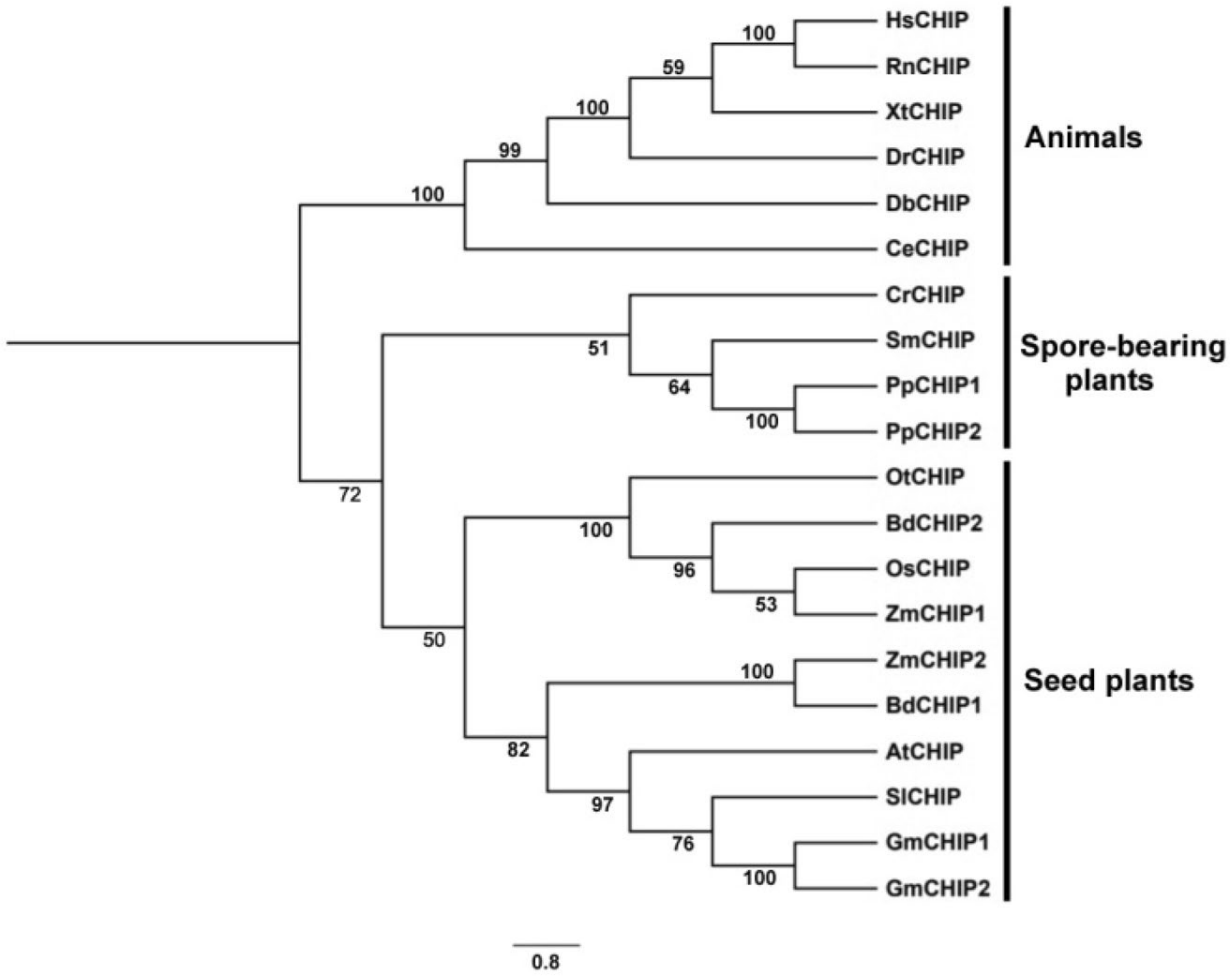
Phylogenetic analysis of CHIP homologs from different species. Twenty CHIP homologs with both TPR motifs and U-box domain from 16 animals and plant species were used in the construction of the phylogenetic tree. The rooted tree was generated with MEGA X v10.0.5 and FigTree v1.3.1 using Neighbor-Joining method with a p-distance model (1000 replicates). Numbers on the tree branches represent bootstrap values.

To analyze the evolutionary relationship of the conserved protein family, we performed phylogenetic analysis of CHIP homologs from different organisms including animals, spore-bearing and seeds plants. As shown in Fig. 2, there are three major clades in the phylogenetic tree. All the CHIP proteins from the animals clustered in one clade, while those from spore-bearing and seed plants clustered in two separate clades (Fig. 2). These results indicate that the topology of phylogenetic tress for CHIP homologs from animals, spore-bearing and seed plants is in the agreement with the evolutionary tree of the organisms. Furthermore, CHIP proteins from monocot and dicot plants also generally clustered separately in the major clade of seed plants (Fig. 2). Interestingly, while the two CHIP homologs from soybean clustered together with CHIP proteins from other dicot plants, the two CHIP homologs from maize and purple false brome have a relative distant relationship (Fig. 2). As expected, one CHIP homolog from each of the two monocot plants clustered with those from other monocot plants but the other homolog was actually grouped closer with CHIP homologs from dicot plants (Fig. 2). The evolutionary significance of the sequence variation of the CHIP homologs from maize and purple false brome is unclear but could reflect potential functional diversification of the two CHIP homologs in these plants.

### Differential induction of *SlCHIP* by heat stress and stress hormones

**Figure 3:**
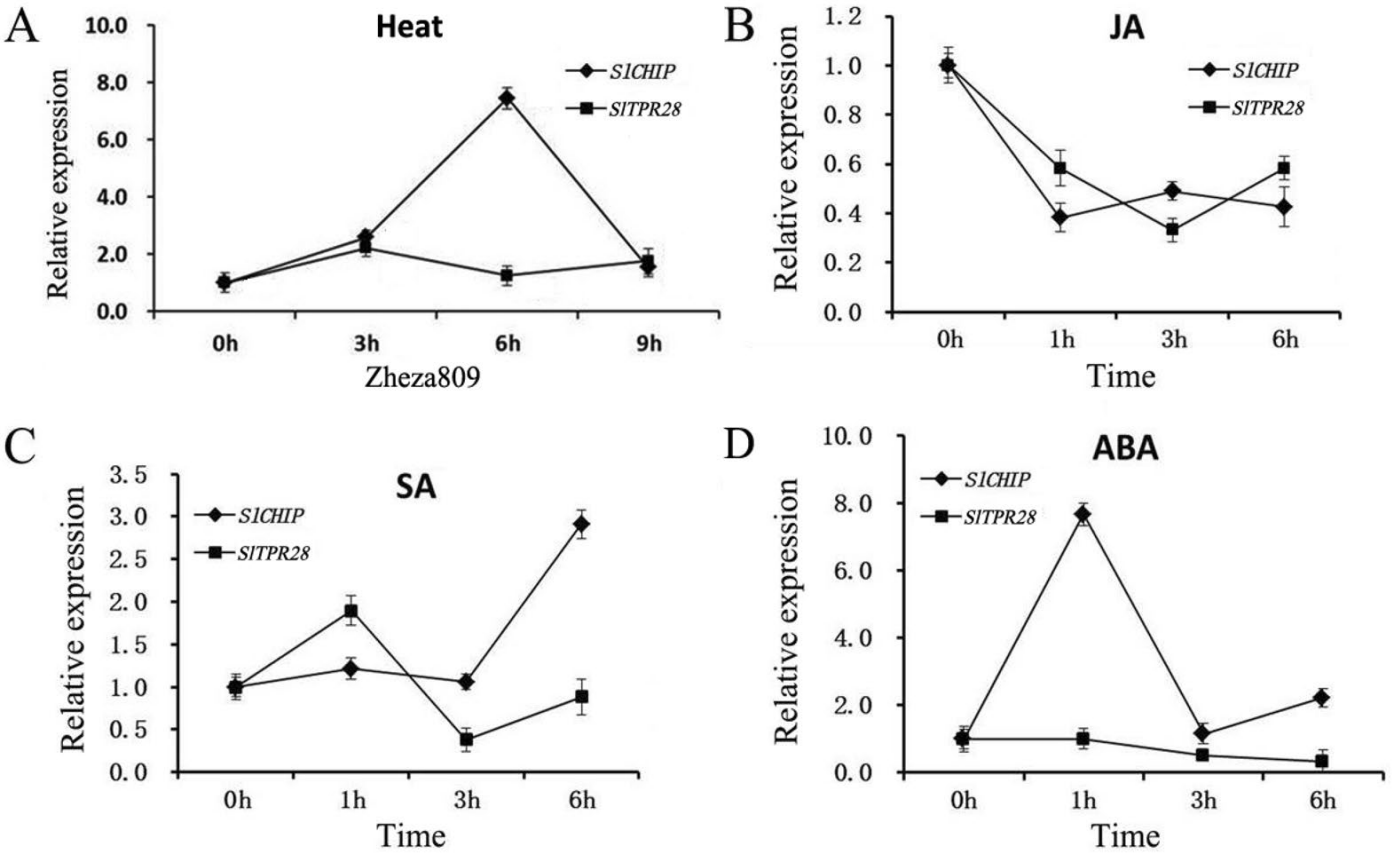
Expression of *SlCHIP* and *SlTPR28* in response to heat, SA, JA and ABA. Six-week-old tomato plants were treated at 45 °C (**A**) or sprayed with 100 μM MeJA (**B**), 20 μM SA (**C**) and 20 μM ABA (**D**) for the indicated amounts of time. At least 3 leaflets of the fourth leaves from 3 individual plants for each sample were collected at 0-, 3-, 6-, and 9-h after treatment for RNA isolation and RT-qPCR analysis. Error bars indicate SE (n = 3). The experiments were repeated three times with similar results.

Arabidopsis mutant plants for CHIP gene *AtCHIP* are compromised in tolerance to a spectrum of abiotic stresses including high temperature but are normal in resistance to the hemibiotrophic bacterial pathogen *Pseudomonas syringe* and the necrotrophic fungal pathogen *Botrytis cinerea*^29^. As a major environmental stress during the whole plant life cycle, heat stress is a major limiting factor for tomato growth and production. In order to explore whether SlCHIP is involved in tomato response to heat stress, we used RT-qPCR to examine expression of the gene after 0-, 3-, 6- and 9-h treatment at 45 °C. For comparison, we included the tomato *SlTPR28* gene in the expression analysis as control. As shown in Fig. 3A, both genes were induced by approximately twofold after 3-h of exposure to the high temperature. Increase in time of exposure to the high temperature caused further increase in the transcript levels of *SlCHIP*, which were more than 7-times higher after 6-h of heat stress (Fig. 3A). After peaking at 6 h of heat treatment, the transcript levels of *SlCHIP* rapidly declined and reached close to the basal levels after 9 h at the high temperature (Fig. 3A). By contrast, no further induction in the transcript levels of *SlTPR28* was detected after the initial increase (Fig. 3A). These results indicated that *SlCHIP* is highly responsive to heat stress.

In plants, different hormones play important roles in diverse biological processes including stress responses. To further examine the involvement of *SlCHIP* and *SlTPR28* in stress responses in tomato, we analyzed their response to JA, SA and ABA, three plant hormones with roles in plant responses to different stresses. SA, JA and ABA were applied to tomato cultivar Zheza809 plants and leaf samples were collected at 0-, 1-, 3- and 6-h after hormone treatments. For comparison, we also analyzed the expression of *AtCHIP* in Arabidopsis Col-0 plants. As shown in Fig. 3B, both *SlCHIP* and *SlTPR28* were down-regulated in their transcript levels by application of exogenous JA. On the other hand, AtCHIP appeared to be significantly induced by JA treatment (Supplemental Fig. S1). Both *SlCHIP* and *SlTPR28* were induced by SA but with different kinetics (Fig. 3C). *AtCHIP* expression was not substantially altered during the first 3 h after SA treatment and displayed significant decline after 6 h of SA treatment (Supplemental Fig. S1). Therefore, there appeared to be significant difference between Arabidopsis and tomato in SA-regulated expression of the homologous *CHIP* genes. On the other hand, there was a strikingly similarity in the ABA-regulated expression of *SlCHIP* in tomato and *AtCHIP* in Araidopsis. Both *CHIP* genes were strongly induced during the first hour after ABA treatment but rapidly declined to the basal levels by the third hour of ABA treatment (Fig. 3D and Supplemental Fig. S1). By contrast, no induction of *SlTPR28* was observed in tomato plants after ABA treatment (Fig. 3D). These results demonstrated that SlCHIP was responsive to plant stress hormones and its strong induction by SA and ABA suggested potential roles in tomato responses to both biotic and abiotic stresses.

### Increased sensitivity of *SlCHIP*-silenced tomato plants to heat stress

**Figure 4:**
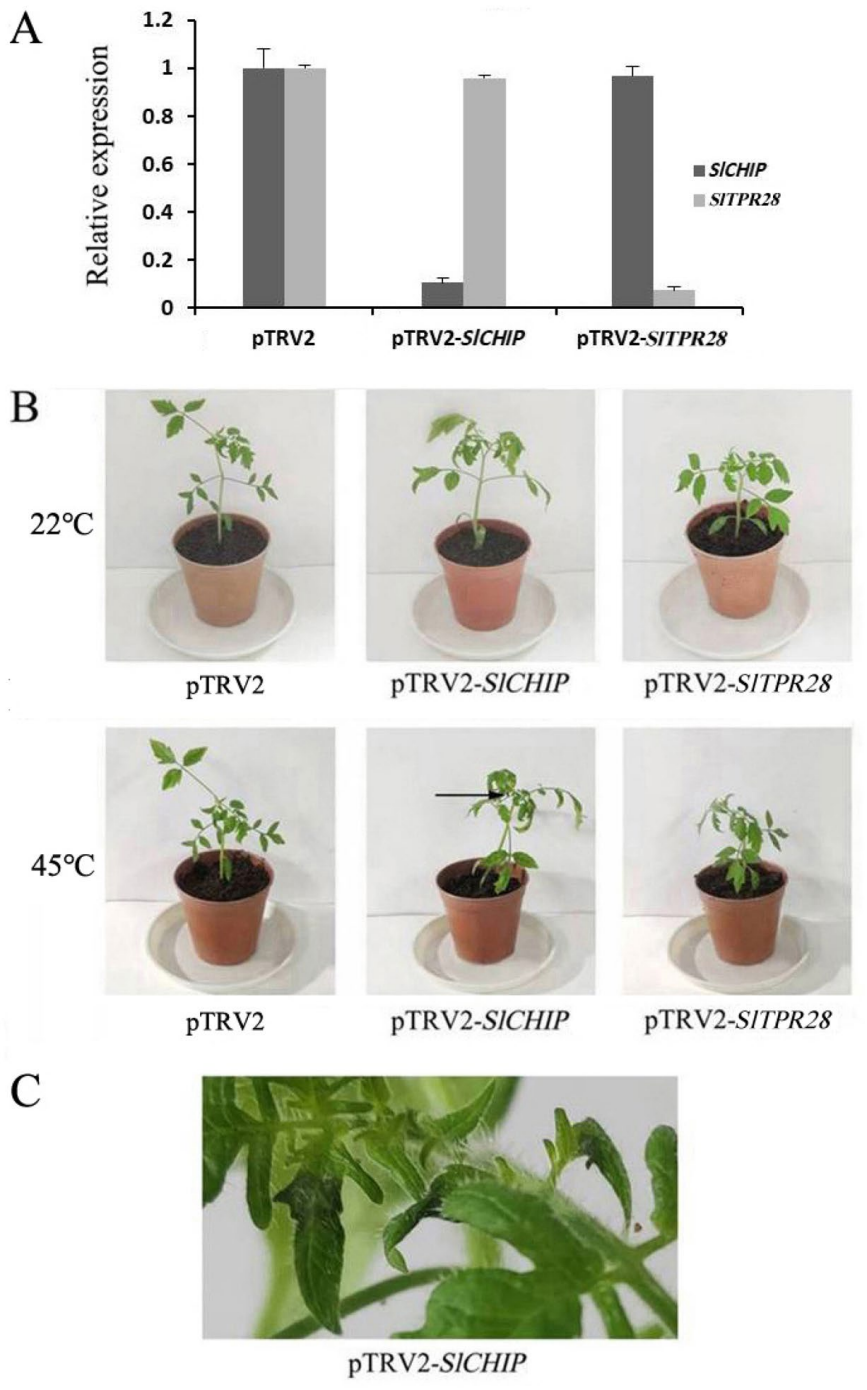
Phenotypic analysis of *SlCHIP*- or *SlTPR28*-silenced plants after heat stress. (**A**) *SlCHIP* and *SlTPR28* transcript levels in *SlCHIP*- and *SlTPR28*-silenced tomato lines. The terminal leaflets in the fifth leaves of 20 individual 6-week-old plants were collected for RNA extraction and RT-qPCR analysis. Means of transcript levels were calculated from 12 plants with > 80% silencing efficiency and shown with SE (n = 12). These plants were used in the subsequent assays of heat tolerance. (**B**) Heat stress symptoms of *SlCHIP-* or *SlTPR28-*silenced plants. Six-weeks old mock (pTRV2), *SlCHIP-*silenced (pTRV2-*SlCHIP*) and *SlTPR28-*silenced (pTRV2-*SlTPR28*) plants were placed in a 45 °C growth chamber for 9 h. The picture was taken after 9 h heat treatment. (**C**) Water soaking symptom on leaflets of the fourth leaves in *SlCHIP-*silenced plants. Experiments were repeated three times with similar results.

In order to address directly the role of SlCHIP in tomato response to heat stress, we conducted VIGS (virus-induced gene silencing) experiments in tomato cultivar Zheza809 to suppress expression of *SlCHIP* and analyze its impact on tomato heat tolerance. A *SlCHIP* gene fragment was cloned into the pTRV2 vector to generate the PTRV2-*SlCHIP* silencing construct, which was coinfiltrated with pTRV1 into tomato plants. Since *SlCHIP* and its only closest homolog *SlTPR28* share 44.9% similarity in nucleic acid sequences, we designed VIGS primers of *SlCHIP* using a 286 bp fragment consisting of 202 bp 5′-UTR and only 84 bp CDS (Supplementary Table S1). As controls, pTRV*2* VIGS constructs for *SlTPR28* (pTRV2-SlTPR28) and tomato PDS gene for phytoene desaturase (pTRV2-PDS), as well as the pTRV2 empty vector (mock) were also coinfiltrated^37^. The bleaching phenotype due to the silencing of the tomato in the pTRV2-*SlPDS* infiltrated plants was used to verify the efficiency of the VIGS procedure. Silencing efficiency was also assessed by testing target gene expression level in the terminal leaflets of the fifth leaves using RT-qPCR with gene-specific primers. The transcript levels of *SlCHIP* and *SlTPR28* in the tomato plants infiltrated with their respective silencing vectors were reduced by more than 80%, when compared to those in pTRV2-infiltrated control plants (Fig. 4A). By contrast, there was no significant cross-silencing of *SlCHIP* in *SlTPR28*-silenced plants or *SlTPR28* in *SlCHIP*-silenced plants as their transcript levels were both similar to those in pTRV2-infiltrated plants (Fig. 4A). These results indicate that TRV-mediated silencing of *SlCHIP* and *SlTPR28* was effective and specific (Fig. 4A).

To determine the effect on tomato heat tolerance, we placed the silenced and control plants in a growth chamber at 45 °C. After 9 h at the high temperature, control plants were very normal with no leaf wilting symptoms, while *SlTPR28*-silenced plants displayed only minor but visible wilty phenotype (Fig. 4B). Importantly, shoots of all *SlCHIP*-silenced plants displayed substantially wilting symptoms after 9-h at the high temperature. More than 80% of leaflets of the fourth leaves in *SlCHIP*-silenced plants were visibly curled (Fig. 4B). Furthermore, approximately 23% of leaflets of the fourth leaves in *SlCHIP1*-silenced plants showed significant water-soaking symptoms and tissue collapse after 6-h at 45 °C (Fig. 4C). These results indicated that SlCHIP played a critical role in tomato heat tolerance.

**Figure 5:**
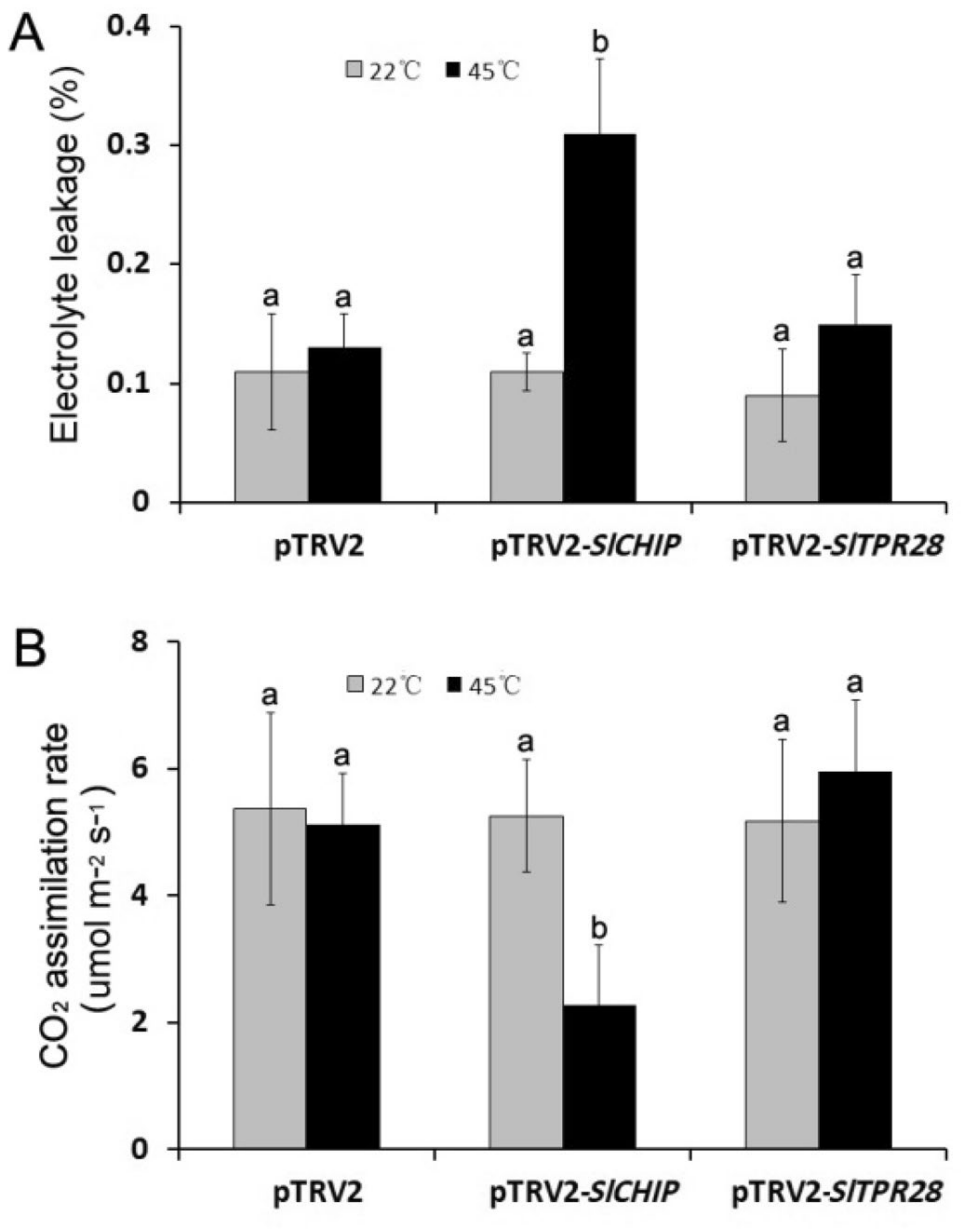
Effects of heat stress on electrolyte leakage and CO_2_ assimilation rate in *SlCHIP*- and *SlTPR28*-silenced plants. (**A**) Electrolyte leakage. (**B**) CO_2_ assimilation rates. Both electrolyte leakage and CO_2_ assimilation rates of the terminal leaflets of the fifth leaves were determined immediately after 9 h at 22 or 45 °C heat treatment. Means and SE were calculated from average values determined from 12 plants per experiment for each type of plants. According to Tukey's multiple comparisons test (*P* = 0.01), means do not differ significantly if they are indicated with the same letter. Experiments were repeated three times with similar results.

To provide more quantitative assessment of the impact of *SlCHIP* silencing on tomato heat tolerance, we compared heat-induced electrolyte leakage in leaves of control and silenced tomato plants. As shown in Fig. 5A, there was a slight but statistically insignificant increase in electrolyte leakage in control or *SlTPR28*-silenced tomato leaves after exposure to 45 °C for 9 h (Fig. 5A), consistent with little development of heat stress symptom in these plants (Fig. 4B). By contrast, there was approximately a twofold increase in electrolyte leakage after heat stress in *SlCHIP*-silenced tomato leaves (Fig. 5A), in agreement with the substantial development of water-soaking and wilting symptoms (Fig. 4). Heat stress causes harmful effects on a variety of biological processes in plants including photosynthesis. Therefore, we also compared *SlCHIP*- and *SlTPR28*-silenced tomato plants with control plants for the effect of heat stress on CO_2_ assimilation rate of the terminal leaflets of the fourth leaves immediately after heat treatment. As shown in Fig. 5B, after 9-h exposure at 45 °C, no statistically significant reduction of CO_2_ assimilation rate was observed in leaves of control or *SlTPR28*-silenced tomato plants. On the other hand, silencing of *SlCHIP* caused more than 55% reduction in CO_2_ assimilation rate after 9-h exposure at 45 °C (Fig. 5B). These results further indicated that silencing of *SlCHIP*, but not *SlTPR28*, substantially compromised tomato heat tolerance.

### Silencing of *SlCHIP* caused increased accumulation of insoluble proteins during heat stress

**Figure 6:**
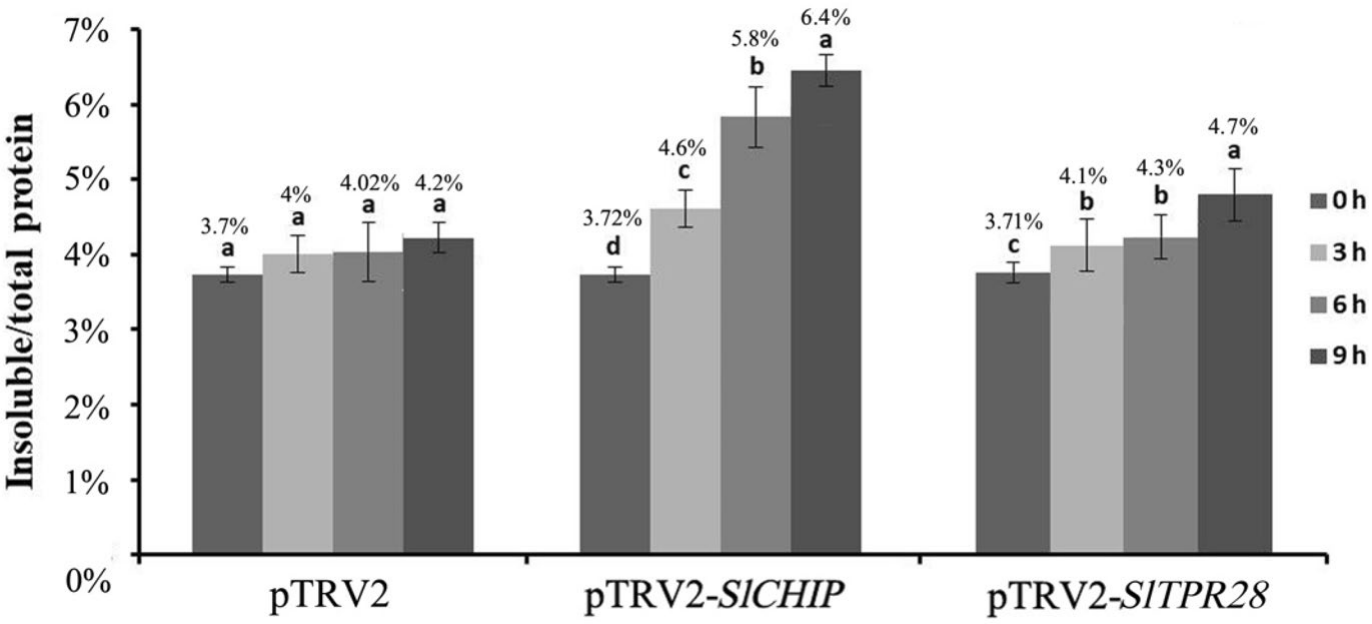
Accumulation of insoluble protein aggregates in *SlCHIP*- and *SlTPR28*-silenced plants under heat stress. Leaf tissues from mock (pTRV2)-, *SlCHIP*-silenced (pTRV2-*SlCHIP*) and *SlTPR28*-silenced (pTRV2-*SlTPR28*) plants were collected at indicated hours at 45 °C for extraction of total, soluble and insoluble proteins as described in Materials and Methods. The starting homogenates as total proteins and the last pellets as insoluble proteins were determined and the percentages of insoluble proteins to total proteins were calculated. Error bars indicate SE (n = 12). According to Tukey's multiple comparisons test (*P* = 0.01), means do not differ significantly if they are indicated with the same letter. Experiments were repeated three times with similar results.

Heat stress usually leads to denatured or damaged proteins and these proteins accumulate as insoluble, detergent-resistant protein aggregates in plant cells^28^. To ascertain whether the compromised heat tolerance of *SlCHIP*-silenced plants was associated with increased accumulation of insoluble, detergent-resistant protein aggregates in plant cells, we isolated and quantified total and insoluble proteins from leaves of control, *SlCHIP-* or *SlTPR28*-silenced plants after 0-, 3-, 6-, and 9-h of heat treatment at 45 °C. As shown in Fig. 6, there was little increase, from 3.7 to 4.2%, in the insoluble proteins as percentages of total proteins in control plants after 9 h at the high temperature. Insoluble proteins as percentages of total proteins also increased marginally (from 3.71 to 4.7%) in *SlTPR28-*silenced plants (Fig. 6). By contrast, in *SlCHIP*-silenced plants, insoluble proteins as percentages of total proteins rose from 3.72 to 6.4%, which represented a 73% increase after 9 h at the high temperature (Fig. 6). Therefore, the increase in insoluble protein aggregates in *SlCHIP-*silenced plants were 5–6 times higher than that in control or *SlTPR28-*silenced plants after 9 h of heat stress. Taken together, these results indicated a critical role of SlCHIP in protection against proteotoxicity by targeting insoluble protein aggregates in tomato leaf cells during heat stress.

### Increased ubiquitination of insoluble protein aggregate in *SlCHIP*-silenced plants

**Figure 7:**
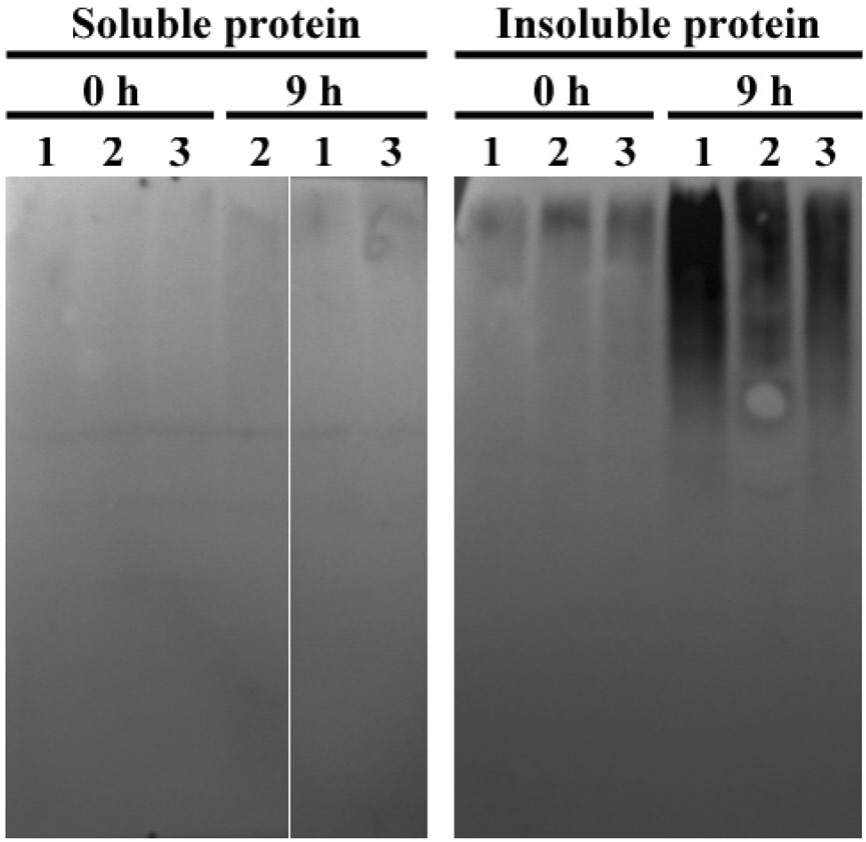
Accumulation of insoluble ubiquitinated proteins in *SlCHIP*- and *SlTPR28*-silenced plants under heat stress. Soluble and insoluble proteins were prepared using low-speed centrifugation from the same amount of total proteins prepared from leaflets of the fourth leaves in *SlCHIP*-silenced (lane 1), *SlTPR28*-silenced (lane 2) and mock (lane 3) plants. The proteins were subjected to SDS-PAGES and probed with anti-ubiquitin monoclonal antibody. The experiment was repeated three times with similar results.

E3 ubiquitin ligase CHIP has been shown to prohibit plastid-destined precursor protein accumulation in the cytosol by regulating precursor degradation through 26S proteasomes^35^. Arabidopsis CHIP mitigates proteotoxicity additively with NBR1-mediated autophagy under heat stress^29^. In order to determine the role of SlCHIP in the ubiquitination of heat-induced insoluble protein aggregates, we compared the extent of ubiquitination of insoluble protein aggregates among control, *SlCHIP*- and *SlTPR28*-silenced plants after 0 and 9 h under heat stress. Soluble and insoluble proteins were isolated, fractionated by SDS-PAGE and assessed for the levels of ubiquitination by proteins blotting using an anti-ubiquitin monoclonal antibody. As shown in Fig. 7 and Supplementary Fig. S2, levels of ubiquitinated protein were all very low in the soluble proteins of all samples with or without heat stress. In the insoluble fractions, the levels of ubiquitination in mock-, *SlCHIP*- and *SlTPR28*-silenced plants were also similarly low without heat stress (Fig. 7 and Supplementary Fig. S3). After 9 h of heat treatment, the levels of ubiquitinated proteins all increased in control, *SlCHIP-* and *SlTPR28*-silenced plants (Fig. 7). However, while the ubiquitinated protein levels in *SlTPR28*-silenced plants were similar to those in control plants, there were clearly higher levels of ubiquitinated proteins in *SlCHIP*-silenced plants after 9 h of heat treatment (Fig. 7). In fact, the levels of ubiquitinated proteins were also elevated even after 1 3, 5, 6 and 7 h of heat stress in *SlCHIP*-silenced plants (Supplementary Figs. S2 and S3). Thus, insoluble proteins accumulated in *SlCHIP-*silenced plants were still highly ubiquitinated under heat stress.

### SlCHIP rescued heat sensitive phenotype of Arabidopsis *atchip* mutants

**Figure 8:**
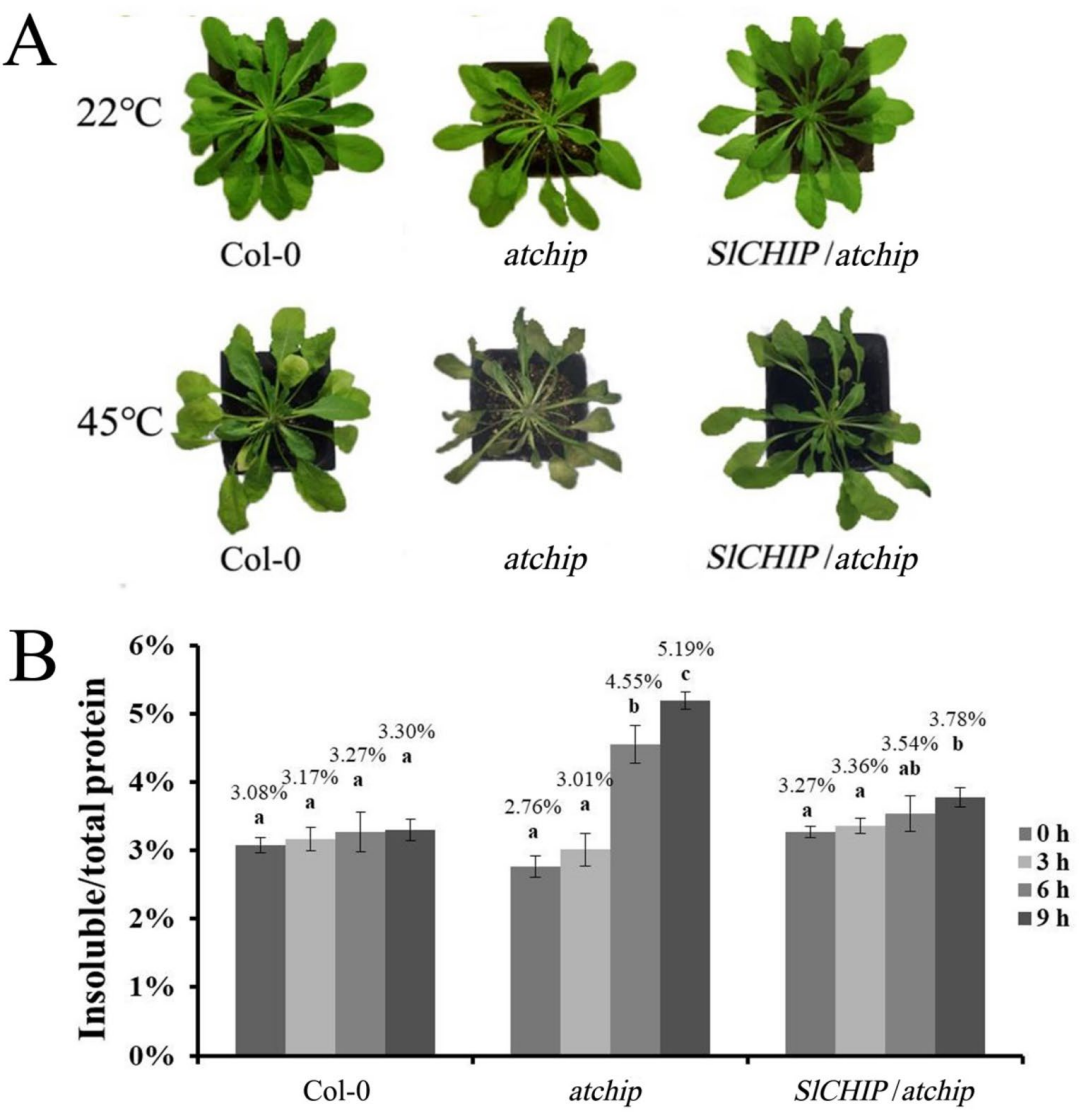
SlCHIP rescued the heat sensitive phenotype of Arabidopsis *atchip* mutant. (**A**) Phenotypic analysis of Arabidopsis wild type Col-0, Arabidopsis *atchip* mutant and transgenic *SlCHIP*/*atchip* plants after 9 h heat stress. Plants were kept in 45 °C. Pictures were taken before and after 9-h heat treatment. (**B**) Accumulation of insoluble protein aggregates. Leaf tissues from Col-0, *atchip* and transgenic *SlCHIP*/*atchip* plants were collected at indicated hours at 45 °C for extraction of total, soluble and insoluble proteins as described in Materials and Methods. The starting homogenates as total proteins and the last pellets as insoluble proteins were used to calculate the percentages of insoluble proteins to total proteins. Error bars indicate SE (n = 6). According to Tukey's multiple comparisons test (*P* = 0.01), means do not differ significantly if they are indicated with the same letter. The experiments were repeated for three times with similar results.

SlCHIP and AtCHIP are 68.8% identical in amino acid sequence and both proteins contain three TPR motifs and one U-box domain. Like *atchip* mutant plants, *SlCHIP*-silenced tomato plants displayed compromised heat tolerance, supporting a conserved role of the E3 ligases in plant heat stress responses. To provide further evidence, we tested whether tomato *SlCHIP* gene could restore the heat tolerance of Arabidopsis *atchip* mutant plants. *SlCHIP* gene was cloned into a plant transformation vector behind the Arabidopsis *CHIP* gene promoter and transformed into an Arabidopsis *atchip* mutant. The transgenic *atchip* mutant lines expressing the *SlCHIP* gene were compared with non-transgenic Col-0 wild type and *atchip* mutants for heat tolerance. Using 12 plants for each genotype and three repeats, we observed wilting and chlorotic symptoms in more than 75% of leaves from the *atchip* mutant plants after 9 h of heat stress (Fig. 8A). However, only 16–20% of leaves of the *atchip* mutant plants expressing *SlCHIP* displayed yellowish and curled symptoms, similar to the 14–18% yellowish and curled leaves in wild type Col-0 plants after exposure for 9 h at 45 °C (Fig. 8A). We also collected leaf samples at 0-, 3-, 6- and 9-h of high temperature for analysis of insoluble protein aggregates. As shown in Fig. 8B, increase in insoluble proteins as percentages of total proteins in *atchip* mutants was nearly 10 times of that in Col-0 wild-type plants. By contrast, insoluble proteins as percentages of total proteins in the transgenic *atchip* mutant plants expressing *SlCHIP* increased only slightly from 3.27 to 3.78% (Fig. 8B). These results demonstrated that *SlCHIP* was able to restore the heat tolerance of the *atchip* mutant plants close to the wild-type levels.

## Discussion

CHIP proteins are present in a large number of eukaryotic organisms including animals and plants^29,36–39^. As a U-box E3 ubiquitin ligase, CHIP collaborates with molecular chaperones for recruitment of misfolded client protein substrates and targets their ubiquitination and degradation by the proteasome system^29,40,41^. As a central component in cellular protein quality control, CHIP has been extensively analyzed in animal systems for its role in a myriad of physiological and pathological processes^37,42–46^. Plants as sessile organisms are constantly exposed to a variety of unfavorable environmental conditions including extreme temperatures and mechanisms for maintaining the integrity of the proteome of plant cells are essential for plant growth, development and survival^47,48^. CHIP proteins are also present in plants but so far there have been only a very few studies on plant CHIP proteins only in Arabidopsis^49–54^. In the present study, we have searched the genomes of a substantial number of spore-bearing and seed plants and identified genes encoding CHIP proteins that contain both TPR motifs at the N-terminus and a C-terminal U-box domain. Like many animal organisms, a majority of plants contain a single CHIP gene, further underscoring the evolutionarily conserved role of the chaperone-dependent E3 ubiquitin ligase in different organisms. In some plants including maize and soybean, there are two genes encoding CHIP proteins most likely due to their polyploid nature with genome duplication during the evolutionary history^55,56^. Interestingly, phylogenetic analysis revealed that while the two CHIP proteins from soybean are highly similar and are both clustered with CHIP proteins from other dicot plants, the CHIP proteins from maize and *Brachypodium distachyon* have diverged significantly in protein sequences and as a result, were placed in different subclades in the phylogenetic tree with one of the two CHIP proteins from each species clustered with those from dicot plants. Whether the structural difference of the CHIP proteins in these plants is associated with functional divergence is unclear but can be addressed through analysis of their expression patterns, biochemical and molecular properties and, most importantly, the phenotypes of their mutants.

As a central E3 ubiquitin ligase in protein quality control, CHIP acts together with chaperones in targeting degradation of misfolded proteins, which are generated at high levels in plant cells under heat stress. To determine the role of tomato CHIP in response to high temperature, we have analyzed the response of tomato *SlCHIP* gene expression to high temperature and discovered it to be heat-inducible (Fig. 3A). Unlike genes encoding HSP proteins with rapid heat induction, increase in the transcript levels of *SlCHIP* was relatively slow but steady during the first 6 h of heat treatment (Fig. 3A). This heat-induced increase in the expression of *SlCHIP* during the early hours of heat stress was followed by a rapid decline after 6 h at the high temperature (Fig. 3A). The early induction of *SlCHIP* during heat stress would lead to elevated levels of *SlCHIP* proteins necessary for the rapid increase in misfolded proteins under the high temperature. The transient nature of the heat-induction of *SlCHIP* expression could be attributed to potential deleterious effect of prolonged or excessive production of the ubiquitin E3 ligase, which could lead to non-specific ubiquitination and degradation of cellular proteins. Consistent with this possibility, overexpression of Arabidopsis *AtCHIP* gene reduces plant tolerance to extreme temperatures^54^. In addition to heat stress, *SlCHIP* was responsive to plant stress hormones JA, SA and ABA. While the responses of *CHIP* to JA and SA appeared to be complex and varied between tomato and Arabidopsis, ABA-induced expression of *CHIP* was rapid and strong in both Arabidopsis and tomato. ABA as an important plant stress hormone plays a critical role in plant responses to heat stress^57,58^. ABA-induced *CHIP* gene expression preceded heat-induced *CHIP* expression and it will be of great interest to determine whether ABA signaling plays a role in heat-induced expression of the *CHIP* genes during plant response to high temperature.

To directly address the role of *SlCHIP* in plant heat tolerance, we have taken two molecular approaches. First, we have successfully demonstrated that expression of *SlCHIP* in the Arabidopsis *atchip* mutant could restore the heat tolerance of the heat-sensitive mutant (Fig. 8), supporting the conserved role of the CHIP proteins from two different plant species in plant heat tolerance. In addition, we employed VIGS to suppress the expression of *SlCHIP* and comprehensively analyzed the impact on tomato heat tolerance. VIGS is rapid, simple and particularly suitable for characterization of phenotypes that might be lethal in stable lines^59^. Potential drawbacks of VIGS include cross-silencing of sequence-related off-target genes. However, there is only a single *CHIP* gene with no close homologs in the tomato genome. In addition, we have included a gene encoding a protein containing TPR motifs as negative control in the study and found no significant cross-silencing among the two related genes based on RT-qPCR analysis of the transcript levels for the genes and the specific nature of the effects of their silencing on the heat tolerance and associated physiological and biochemical parameters.

Virus-induced silencing of *SlCHIP* caused increased water-soaking and wilting symptoms in tomato plants after incubation at 45 °C, which were much less in *SlTPR28*-silenced plants and barely observed in control plants infiltrated with the pTRV2 empty vector. These observations provided direct and compelling evidence for a critical role of *SlCHIP* in tomato heat tolerance. Increased development of heat stress symptoms in the *SlCHIP*-silenced tomato plants was associated with elevated electrolyte leakage (Fig. 5A), which is a hallmark of stress-induced injury of plant cells associated with reduced integrity of cell membrane, increased production of ROS and programmed cell death^60^. Furthermore, we observed reduced capacity of photosynthesis in the leaves of *SlCHIP*-silenced but not in *SlTPR28*-silenced or control tomato plants after exposure to high temperature (Fig. 5B). Chloroplasts are one of the most sensitive organelles in plants to heat stress and apparently CHIP has a critical role in protection of chloroplasts from heat stress even though the heat-regulated E3 ubiquitin ligase is mostly present in the cytosol. Previously, it has been shown that in Arabidopsis, CHIP and HSC70 mediate plastid-destined precursors degradation by the proteasome system^35^. These proteins are synthesized in the cytosol as unfolded precursors and can accumulate in the cytosol under conditions when their import into plastids is hindered^35^. These protein precursors, if not promptly degraded, can accumulate in the cytosol to form nonspecific aggregates and cause severe cellular damage. Under high temperatures, denatured proteins increase and the capacity of cellular protein quality control will be under a great pressure. Under such conditions, maintaining plastid-destined protein precursors at unfolded conformation may become increasingly insufficient, leading to their misfolding and formation of protein aggregates. Reduced levels of CHIP proteins as in *SlCHIP*-silenced tomato plants would compromise the ability to degrade these misfolded proteins or protein aggregates, leading to toxic effects on cellular structures including the photosynthetic organelles of plant cells.

As a chaperone-dependent E3 ubiquitin ligase, CHIP targets ubiquitination of misfolded proteins for degradation by the 26S proteasome system. In the absence of CHIP, these misfolded proteins will form protein aggregates, which could be targeted for degradation by other degradative pathways such as selective autophagy^29^. Indeed, increased heat sensitivity in *SlCHIP*-silenced tomato plants was associated with elevated levels of protein aggregates (Fig. 6). Interestingly, protein blotting using an anti-ubiquitin monoclonal antibody showed that the protein aggregates accumulated in *SlCHIP*-silenced tomato plants were still highly ubiquitinated (Fig. 7). One possible explanation for the counterintuitive observation is that SlCHIP specifically or preferentially recognize those misfolded but still soluble proteins through their associated chaperone proteins and targets then for ubiquitination and degradation by the proteasome system. Those soluble misfolded proteins, if not promptly degraded, will then interact nonspecifically to form insoluble protein aggregates. Apparently there are additional ubiquitin E3 ligases that recognize these misfolded proteins during or after their aggregation for ubiquitination and ultimately degradation by selective autophagy or other pathways. Therefore, it will be of importance to identify and analyze the unknown E3 ligases and associated protein degradation pathways that function coordinately in the recognition, ubiquitination and processing of stress-induced abnormal proteins that are generated under stress conditions. In addition, a large number of studies in animals have established that CHIP E3 ligases have important roles not only in protein quality control but also in signaling through targeting of specific regulatory proteins^37–39,43,45^. It is highly conceivable that CHIP may have a similar signaling role in plant stress responses. A comprehensive analysis of the roles and mode of action of CHIP proteins will provide important new insights into the molecular link between protein quality control networks and plant stress responses.

## Methods

### Plant materials and growth conditions

The tomato cultivar Zheza809 was used in this study. Arabidopsis T-DNA insertion mutant *atchip-1* (Salk_048371) in Col-0 accession has been previously described^29^. Tomato and Arabidopsis plants were grown in a growth room at 22–24 °C on a photoperiod of 12-h light (600 µmol m^−2^ s^−1^) and 12-h dark. Heat treatment was performed by placing plants in a growth chamber at 45 °C with 200 µmol m^−2^ s^−1^ light for 9 h to test heat tolerance.

### Cloning of *SlCHIP* and *SlTPR28* and phylogenetic analysis

Total RNA was isolated from plants and reverse-transcribed using RNAsimple Total RNA Kit (DP419) and FastKing gDNA Dispelling RT SuperMix(KR118)(Tiangen, Beijing, China) respectively, according to the manufacturer’s recommendations. The cDNA was used as template for PCR amplification of the CDSs of *SlCHIP* and *SlTPR28* using gene-specific primers (Supplementary Table S1). The CDSs of *SlCHIP* and *SlTPR28* were cloned into pMD18-T vector, verified by sequencing and used for the subsequent experiments.

CHIP protein sequences from animals were identified from NCBI. The predicted protein sequences of tomato SlCHIP and Arabidopsis AtCHIP were used to conduct blast search to identify plant CHIP homologs through Phytozome, the Plant Comparative Genomics portal of the Department of Energy's Joint Genome Institute (https://phytozome.jgi.doe.gov/pz/portal.html). CHIP homologs from plant species were selected to perform phylogenetic analysis. Accession number of animal and transcript name of plant CHIP homologs were summarized in Supplementary Table S2. Lasergene, MEGA X v10.0.5 and FigTree v1.3.1 softwares were used to execute sequence alignment and generate phylogenetic tree.

### RT- qPCR analysis of gene expression in response to plant hormones

To analyze gene expression in response to hormones, we treated 6-week-old tomato and 4-week-old Arabidopsis plants by foliar spraying with 20 μM ABA, 20 μM SA, 100 μM methyl jasmonate (MeJA) or water as a control. Samples were collected at 0-, 1-, 3- and 6-h after treatment. Total RNA and cDNA were prepared as described earlier. RT-qPCR was performed using the CFX96 Touch Real-Time PCR Detection System (Bio-Rad, CA, USA) and SYBR Premix Ex Taq Kit (TaKaRa, Dalian, China). The tomato *SlACTIN7* gene and the *Arabidopsis AtACTIN2* were used as internal controls. Three replicates were used for treatment and the experiments were repeated three times. The relative gene expression was calculated using 2^−ΔΔC^_T_ method^61^. Gene-specific primers for qRT-PCR are listed in Supplementary Table S1.

### Virus-induced Silencing of *SlCHIP* and *SlTRP28*

*SlCHIP* and *SlTPR28* fragments of 286 and 364 bp, respectively, were amplified and cloned into vector pTRV2 to generate pTRV2-*SlCHIP* and pTRV2-*SlTPR28*, respectively. Plasmids pTRV2, pTRV2-*SlCHIP* and pTRV2-*SlTPR282* were transformed into *Agrobacterium tumefaciens* strain GV3101 competent cells by electroporation and transformants were selected on YEB plates containing kanamycin (50 ng/ml), rifampicin (50 ng/ml) and gentamycin (25 ng/ml). Plates were kept at 28 °C in an incubator for 48 h and positive colonies were confirmed by colony PCR. Agrobacteria carrying pTRV2, pTRV2-*SlCHIP* or pTRV2-*SlTPR28* were used to perform VIGS according to the protocol as described^62^. Infiltration was performed on 30 tomato plants for each gene and the experiments were repeated three times. RT-qPCR was performed to determine the silencing efficiency for each gene using RNA isolated from the terminal leaflets of the fifth leaves. Only those tomato plants with more than 80% reduction in the transcript levels for the silenced gene were used in the subsequent assays for heat tolerance.

### Assays of heat tolerance

Selected 6-week old tomato and 4-week old Arabidopsis plants were placed in a growth chamber at 45 °C with 200 µmol m^−2^ s^−1^ light for 9 h to test heat tolerance. Three replicates were performed with 12 plants for each replicate for every type of plants. Tomato leaf samples were collected with 0-, 3-, 6- and 9-h high temperature. Heat stress symptoms including wilting and water soaking were observed at indicated times by taking imaging of treated plants or counting of symptomatic leaves.

Determination of electrolyte leakage caused by high temperature was performed after heat stress as previous described^63^. Briefly, seven leaf disks from the leaflets of the fourth and fifth leaves were sampled, rinsed with deionized water and put into tubes with 20 ml deionized water for 20 h at 24 °C. The conductivity was measured before and after tubes autoclaved using a potable MW802 pH/EC/TDS meter (Milwaukee Instruments, Inc., Rocky Mount, NC, USA). The CO_2_ assimilation rates of the terminal leaflets of the fourth leaves were determined in the silenced and pTRV2 plants with an infrared gas analyzer-based potable photosynthesis system (LI-6400; Li-COR, Lincoln, NE, USA).

### Generation of *SlCHIP* transgenic *Arabidopsis* lines

In order to generate transgenic *SlCHIP*-expressing Arabidopsis lines, the full length coding sequence for *SlCHIP* gene was amplified from plasmid pMD18-*SlCHIP* and inserted into the plant transformation vector pFGC5941-3ΧHA with the 1.5 Kb Arabidopsis *CHIP* gene native promoter from Arabidopsis Col-0 plant. The resulting plasmid was confirmed by colony PCR and transformed into *atchip-1* mutant plants (Salk_048371). Transformants were screened by selecting for resistance to Basta. Transgenic plants expressing *SlCHIP* were determined by western blot using an anti-HA monoclonal antibody. Homozygous T2 transformants were selected and used in the study.

### Soluble and insoluble proteins extraction and western blotting

Tomato and *Arabidopsis* leaf samples were collected at 0-, 3-, 6- and 9-h after heat treatment. Leaf samples were ground in liquid nitrogen and homogenized in a detergent containing extraction buffer as described previously^29^. Total proteins were first passed through filtration to remove debris and soluble and detergent-resistant insoluble proteins were separated by low speed centrifugation as previously described^28^. The protein quantity was measured using BCA (Bicinchoninic acid) Protein Assay Kit (SK1070, Coolaber, Beijing, China) based on the manufacturer’s instruction. Ubiquitinated proteins were detected by western blotting with an anti-ubiquitin monoclonal antibody (Sigma, USA). The antigen–antibody complexes were detected by enhanced chemiluminescence using luminal as previously described^28^.

## Supplementary Information

Supplementary Information
