# Supplementary material for: Functional analysis of tomato CHIP ubiquitin E3 ligase in heat tolerance
Source: Sci Rep. 2021 Jan 18;11:1713. doi: 10.1038/s41598-021-81372-8 (PMC7814054; doi:10.1038/s41598-021-81372-8)
Supplement: Supplementary file 1 — Supplementary Information [file 41598_2021_81372_MOESM1_ESM.pdf]

Functional Analysis of Tomato CHIP Ubiquitin E3 Ligase in Heat Tolerance

Yan Zhang[a,*], Xiaodong Lai[a], Siqing Yang[a], Huan Ren[a], Jingya Yuan[a], Huanchun Jin[a], Chengchen Shi[a], Zhibing Lai[b], Gengshou Xia[a]

Includes

Supplementary Figure 1. Expression pattern of *AtCHIP* in response to exogenous SA, JA and ABA.

Supplementary Figure 2. Ubiquitinated protein levels of soluble and insoluble proteins from *SlCHIP*-, *SlTPR28*-silenced and mock plants before and after heat stress.

Supplementary Figure 3. Increased insoluble ubiquitinated proteins in the *SlCHIP*-silenced plants exposed to heat stress.

Supplementary Table 1. List of primers used in this study.

Supplementary Table 2. Accession numbers or transcript names of CHIP homologs from 18 animal and plant species.

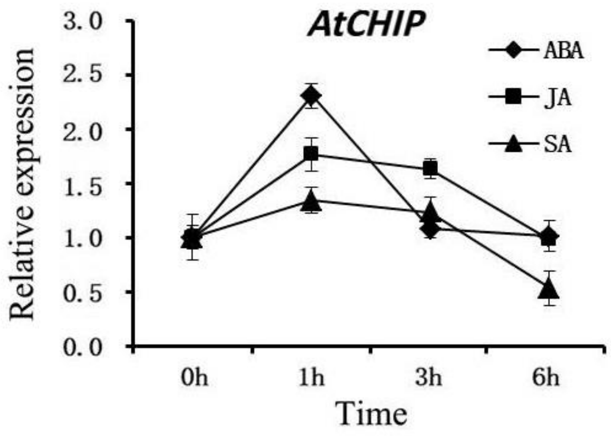

Supplementary Figure 1. Expression of *AtCHIP* in response to exogenous SA, JA and ABA. Four-week-old Arabidopsis Col-0 plants were treated with 20 μM SA, 100 μM methyl jasmonate (MeJA) and 20 μM ABA. At least 3 leaves from 3 individual plants for each sample were collected at indicated hours after treatment. Error bars indicate SE (n = 3). The experiment was repeated three times with similar results.

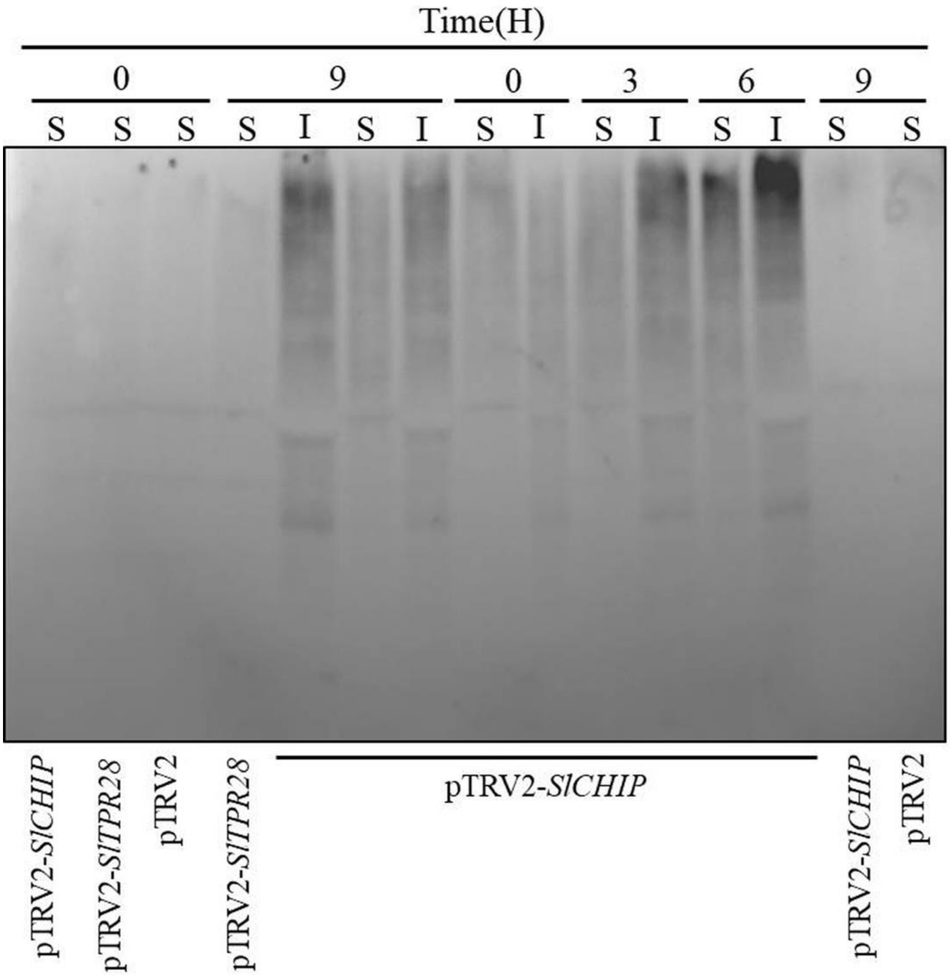

Supplementary Figure 2. Ubiquitinated protein levels of soluble and insoluble proteins from *SlCHIP*-, *SlTPR28*-silenced and mock plants before and after heat stress. The first supernatants as soluble protein and the last pellets as insoluble protein were subjected to SDS-PAGES and probed with anti-ubiquitin monoclonal antibody. The experiment was repeated three times with similar results. Samples from *SlCHIP*-silenced plants after heat treatment for 3- and 6-hours were also collected and ubiquitinated levels of soluble and insoluble proteins from these samples were checked. S, soluble. I, insoluble.

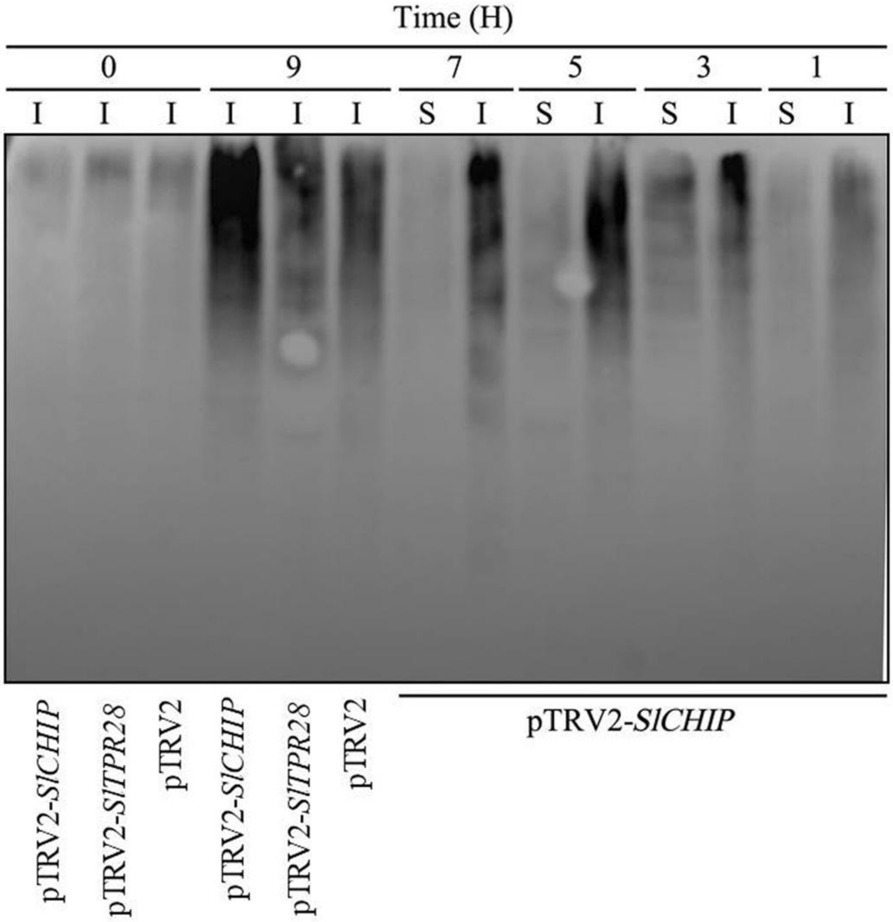

Supplementary Figure 3. Increased insoluble ubiquitinated proteins in the *SlCHIP*-silenced plants exposed to heat stress. The first supernatants as soluble protein and the last pellets as insoluble protein were subjected to SDS-PAGES and probed with anti-ubiquitin monoclonal antibody. The experiment was repeated three times with similar results. Samples from *SlCHIP* silenced plants after indicated hours of heat treatment were also collected and ubiquitinated levels of soluble and insoluble proteins from these samples were checked. S, soluble. I, insoluble.

Supplementary Table 1. List of primers used in this study.

Primers	Sequences (5'-3')	Size (bp)	Purpose
**Cloning**			
SlCHIP-C-F	ATGGCACCAATCGTGGGTTCAAAG	831	To gain 831 bp CDS of *SlCHIP* for construct pMD18-T- SlCHIP1.
SlCHIP-C-R	CTATATCCTATATGCCCAGCCAT		
SlTPR28-C-F	ATGGAGCTCGCCTTGGGGGCCAT	957	To gain 957 bp CDS of *SlTPR28* for construct pMD18-T- SlCHIP2.
SlTPR28-C-R	CTATTCTTGCTTGAATACACGCG		
**VIGS**			
SlCHIP-V-F	GGGGTACC(*Kpn*I)TTCTGCTCCTTCCGCAC	286	To gain 286 bp fragment of *SlCHIP* mRNA for construct pTRV2-*SlCHIP1*.
SlCHIP-V-R	CCCTCGAG(*Xho*I)AGCGCCAAACCGATTCT		
SlTPR28-V-F	GC TCTAGA(*Xba*I)ATACGAGGATGCCTTGG	364	To gain 364 bp fragment of *SlTPR28* CDS for construct pTRV2-*SlTPR28*
SlTPR28-V-R	C GAGCTC(*Sac*I)AGCTGGAGCGTACTTTT		
**Transgenic**			
SlCHIP-T-F	ATGGATCC(*Bam*HI)ATGGCACCAATCGTGGGTTC	847	To gain 831 bp CDS of SlCHIP for construct pFGC5941-*SlCHIP-3HA*.
SlCHIP-T-R	ATCTCGAG(*Xho*I)TATCCTATATGCCCAGCCATG		
**qRT-PCR**			
SlCHIP-Q-F	TACTTTGTGCCCTAATGTTCCGATA	159	To test SlCHIP transcript level in VIGS plants or after treatment with SA, JA and ABA.
SlCHIP-Q-R	TGTAGCAATGCAAGACCAAGATAAT		
SlTPR28-Q-F	ACCCATCAAACGCCACTCTTTACAG	171	To test SlTPR28 transcript level in VIGS plants or after treatment with SA, JA and ABA.
SlTPR28-Q-R	CATCCTCGTATCGTTCCATAGCCTC		
SlACTIN7-Q-	TGAGCTTCGAGTTGCTCCTGA	144	To be used as reference gene.
SlACTIN7-Q-	AGCACAGCCTGGATAGCAACA		
AtCHIP-Q-F	TTTGGTGCTGCCATTGATGC	175	To test *AtCHIP* transcript level after treatment with SA, JA and ABA.
AtCHIP-Q-R	GCATGTAGTGTGCCTTGACG		
AtACTIN2-F	GCTGTTGACTACGAGCAGGA	143	To be used as reference gene.
AtACTIN2-R	ACAAACGAGGGCTGGAACAA		

Supplementary Table 2. Accession numbers (No.) or transcript names of CHIP homologs from 16 animal and plant species.

Species	CHIP homolog	No. or Name	Species	CHIP homolog	No. or Name
**Animals**					
Homo sapiens	HsCHIP	AAD33400	*Danio rerio*	DrCHIP	NP_955968
Caenorhabditis elegans	CeCHIP	NP_491781	*Drosophila busckii*	DbCHIP	XP_017839474
Rattus norvegicus	RnCHIP	NP_001020796	*Xenopus tropicalis*	XtCHIP	NP_001072347
**Plants**					
Arabidopsis thaliana	AtCHIP	AT3G07370.1	*Glycine max*	GmCHIP1	Glyma.03G088400.1
Chlamydomonas reinhardtii	CrCHIP	Cre11.g479650.t1.2	*Glycine max*	GmCHIP2	Glyma.U008400.1
Brachypodium distachyon	BdCHIP1	Bradi3g13620.1	*Oropetium thomaeum*	OtCHIP	Oropetium_20150105_15229
Brachypodium distachyon	BdCHIP2	Bradi2g39800.1	*Oryza sativa*	OsCHIP	LOC_Os05g01460.1
Physcomitrium patens	PpCHIP1	Pp3c14_2850V3.7	*Selaginella moellendorffii*	SmCHIP	Selaginella74711
Physcomitrium patens	PpCHIP2	Pp3c10_2570V3.5	*Solanum lycopersicum*	SlCHIP	Solyc06g083150.2.1
Zea mays	ZmCHIP1	Zm00008a023978_T01	*Zea mays*	ZmCHIP2	Zm00008a023494_T01
